# The relationship between two types of impaired emotion processing: repressive coping and alexithymia

**DOI:** 10.3389/fpsyg.2015.00809

**Published:** 2015-06-16

**Authors:** Lynn B. Myers, Nazanin Derakshan

**Affiliations:** ^1^College of Health and Life Sciences, Brunel University, Uxbridge, UK; ^2^Department of Psychology, Birkbeck, University of London, London, UK

**Keywords:** repressive coping style, alexithymia, interviews, questionnaires

## Abstract

The constructs of repressive coping and alexithymia are both related to impaired emotion processing, yet individuals with a repressive coping style (repressors) score lower than controls on standard self-report measures of alexithymia. A large body of evidence indicates that repressors avoid negative affect. Therefore, the current study examined the relationship between repressive coping and alexithymia by using independently-rated interviews with the aim of bypassing repressors’ tendency of avoiding negative affect. Results showed that repressors scored high on alexithymia, similar to anxious individuals on the independently-rated interview, but scored low on alexithymia on a questionnaire measure. Our findings confirm a link between alexithymia and repressive coping and stress the need for non-standard measures in exploring the nature of the relationship between repressive coping and alexithymia.

## Introduction

It is over 30 years since Weinberger et al. (1979) identified individuals who possess a repressive coping style. Since then numerous studies have demonstrated that “repressors” avoid negative affect, using an avoidant style of processing negative information with a capacity to avoid socially threatening information (see Myers, 2000, 2010; for reviews). One of the defining characteristics of repressors is that they report low levels of subjective distress during potentially stressful situations although they exhibit high physiological and behavioral arousal. This “repressive dissociation” was noted in the original Weinberger et al. (1979) study and is a widely replicated finding (e.g., Derakshan and Eysenck, 1997; Lambie and Baker, 2003). According to vigilance-avoidance theory (Derakshan et al., 2007) repressors have an initial rapid vigilant response triggering behavioral and physiological responses followed by an avoidance stage involving avoidant cognitive biases that inhibit the conscious experience of anxiety. Results from a fairly recent fMRI study were consistent with this theory, as the repressive coping style appeared to be associated with increased brain activity in response to threatening compared to neutral facial expressions at an automatic, non-conscious stage of information processing (Paul et al., 2012).

Research also indicates that having a repressive coping style is associated with poor physical health (see Myers et al., 2007; Myers, 2010; for reviews). Repressors are an important group to understand as they comprise up to 20% of non-clinical populations and up to 50% of chronic illness groups and the elderly (e.g., Erskine et al., 2007; Prasertsri et al., 2011).

Weinberger et al. (1979) identified repressors by their low scores on trait anxiety and high scores on defensiveness. Apart from the repressor group, three control groups are usually identified using the same classification: a further low trait anxiety group who is low on defensiveness (low-anxious) and two high trait anxiety groups, one of which is low on defensiveness (high-anxious) and one which is high on defensiveness (defensive high-anxious).

Similar to repressive coping, alexithymia is a construct which involves difficulties in expressing emotions. Individuals high on alexithymia have difficulties distinguishing emotions from bodily sensations (see Lumley et al., 2007, for a review). Unlike repressive coping which is often seen as a defense, alexithymia is fundamentally a developmental disorder. It literally means “no words for emotion” (Sifneos, 1972). According to more recent formulations of this construct, individuals high on alexithymia are seen to have deficits in cognitive processing and emotional regulation (Taylor et al., 1997). Recently, it has been concluded that a further understanding of alexithymia requires a combined developmental, neuropsychological and biological perspective (Wingbermuhle et al., 2012). Some research indicates an association between alexithymia and physical illnesses (see Lumley et al., 2007, for a review), although the evidence is mixed, possibly due to limitations in popular measures of alexithymia.

It can be concluded that both repressive coping and alexithymia are associated with deficits in emotional processing. Both constructs have produced large bodies of literature (see Lumley et al., 2007; Myers, 2010, for reviews). Although it has been suggested that repressive coping and alexithymia are related constructs (Bonanno and Singer, 1990; Lane et al., 2000) and that individuals who possess a repressive coping style will also be high on alexithymia, this prediction has not been systematically investigated.

Using the Weinberger et al. (1979) classification, it can be predicted that repressors will score high on alexithymia, compared to the truly low anxiety group (low-anxious). However, no predictions can be made concerning any differences between repressors and the two high anxiety groups (high-anxious and defensive high-anxious) as a considerable number of studies indicate that anxiety is positively correlated with alexithymia (see Lumley et al., 2007).

However, two studies that have compared the two constructs using a widely used questionnaire measure of alexithymia (the Toronto Alexithymia Scale, TAS, Taylor et al., 1985; or the revised version, TAS-20; Bagby et al., 1993) have found that repressors score low on alexithymia compared to control groups (Newton and Contrada, 1994; Myers, 1995). Newton and Contrada (1994) found that repressors scored significantly lower on alexithymia than the high-anxious using the original TAS. Myers (1995), using the TAS-20, reported that repressors scored significantly lower than the low-anxious and the two high trait anxiety groups. However, the findings from these two studies may be due to the nature of the instruments used, as a large body of research indicates that repressors avoid negative information about themselves especially in the case of standard self-report measures such as questionnaires (see Myers, 2000, 2010; for reviews).

Yet research has indicated there are limits to repressors’ avoidance of negative affect. It may be possible to bypass repressors’ avoidance in reporting negative affect by using measures which allow independent raters to judge participants’ responses, rather than participants rating their own experiences. Evidence to support this was found in investigations of repressors’ childhood experiences. Using a semi-structured interview for assessing early experiences which utilized independent raters, repressors reported a significantly more negative view of their fathers than non-repressors, whereas using standard questionnaire measures of childhood experiences, repressors reported a significantly more positive view of their fathers than non-repressors (Myers and Brewin, 1994; Myers, 1999; Myers et al., 1999). It can be concluded that this lack of concordance between results of independently- rated interviews and self-report measures highlight the importance of investigating other constructs using a similar methodology to bypass repressors avoidance of negative affect. Consequently, in the current study by using interviews and independent raters to judge whether repressors are high or low on alexithymia, we investigated whether this method would indeed bypass repressors’ avoidance of negative affect and provide a more accurate measure than questionnaires.

In the current study we measured alexithymia with an independently-rated measure, the Observer Alexithymia Scale (OAS, Haviland et al., 2000), where independent raters (usually friends, family or therapists) rate individuals on items which comprise alexithymia. We investigated whether the use of the OAS could be extended to asking raters who have not met the participants to rate participant style by listening to an interview not related to alexithymia.

We included a standard questionnaire measure of alexithymia, the TAS-20 as a reference point to replicate previous findings in this area (e.g., Newton and Contrada, 1994; Myers, 1995). We expected a dissociation between self-report and an independently-rated measure of alexithymia. Supporting this prediction, a previous study highlighted a discrepancy between interviewer ratings of emotional experiences and self-reports on the TAS-20 (Leising et al., 2009). We hypothesized that compared to the truly low-anxious, repressors would score significantly higher on alexithymia on an independently- rated measure of alexithymia, the OAS, but significantly lower than the low-anxious on a questionnaire measure, the TAS-20. As anxiety and alexithymia are positively correlated, no differences were hypothesized between repressors and the two high trait anxiety groups (high-anxious and defensive high-anxious) for the independently- rated OAS, but it was hypothesized that consistent with previous studies (Newton and Contrada, 1994; Myers, 1995) repressors will score significantly lower than the high anxiety groups on the TAS-20.

## Materials and Methods

### Participants

Fifty-six undergraduate students from a UK University participated in the study. They were chosen from an initial pool of 145 students, as part of a larger study on repressive coping, childhood experiences and romantic adult attachment styles, funded by the British Academy. The study obtained ethical approval from Brunel University, UK. There were 45 women and 11 men, aged between 18 and 30 years, with a mean age of 22.47 years (SD = 2.43). Participants were selected on the basis of their scores on the Marlowe-Crowne Social Desirability Scale (MC; Crowne and Marlowe, 1964), a measure of defensiveness, and the Bendig short form of the Taylor Manifest Anxiety Scale (MAS; Bendig, 1956), a measure of trait anxiety. Classification of participants based on the original Weinberger et al. (1979) definition typically divides individuals into four groups: Repressors (low anxiety–high defensiveness); low anxious (low anxiety–low defensiveness), high anxious (high anxiety–low defensiveness), and defensive high anxious (high anxiety–high defensiveness).

Extreme scores on the MC/MAS were identified using the same cut-off points as Vetere and Myers (2002). Repressors (R, *N* = 15), scored 7 or under on the MAS and 17 or over on the MC, low-anxious (LA, *N* = 13), scored 7 or under on the MAS and 11 or under on the MC, high-anxious (HA, *N* = 14) scored 12 or over on the MAS and 11 or under on the MC and defensive high-anxious (DHA, *N* = 14) scored 12 or over on the MAS and 17 or over on the MC.

### Measures

The (OAS, Haviland et al., 2000) is a 33-item observer-rated measure, consisting of statements rated on a 4-point Likert scale which are scored on how like/not like the person is on each statement: e.g., “is a warm person” and “falls apart when things are really tough.” There are five subscales: distant, uninsightful, somatizing, humorless, and rigid.

Ratings on the OAS were made by two independent raters (LM, RE) after listening to a recorded interview (approximately 90 min) consisting of two semi-structured interviews: a parenting interview (see Myers and Brewin, 1994) and a brief romantic attachment interview (BRAI), developed for a study on repressive coping, childhood experiences and romantic attachment styles. Raters were blind to participants’ MAS/MC scores. Before the study, raters underwent training by listening to and scoring pilot study interviews and discussing their OAS ratings in depth, until high agreement was achieved.

Six participants refused to be interviewed, therefore 50 participants were rated on the OAS: R = 14, LA = 13, HA = 13, DHA = 10. Because these interviews do not have any information about physical health, the somatizing scale was omitted.

Pearson correlations between raters were calculated for each scale. Correlations were good (distance *r* = 0.87, *p* < 001; uninsightful *r* = 0.79, *p* < 0.001; humorless *r* = 0.90, *p* < 0.001 and rigid *r* = 0.72, *p* < 0.001). Due to the high correlations between raters, the findings were averaged and that index is reported. Cronbach’s alpha scores for the four scales were very good: distant, 0.95; uninsightful, 0.82; humorless, 0.97; and rigid, 0.87.

TAS-20 (Bagby et al., 1993) is a 20-item measure consisting of statements with which participants are instructed to rate how much they agree or disagree on a five-point Likert scale. Examples of items are “It is difficult for me to find the right words for my feelings” and “when I am upset, I don’t know if I am sad, frightened, or angry.” In the present study, Cronbach’s alpha for the overall scale was 0.80.

### Procedure

Participants completed the TAS-20 along with a number of other questionnaires at least 2 days before an individual testing session where they were interviewed. Interviews took place in a quiet lab and were recorded for later analyses.

## Results

Group differences in the means were tested for using one-way analysis of variance (ANOVA). Newman-Keuls tests were used for *post hoc* comparisons, with significance levels set at *p* < 0.05.

### OAS

**FIGURE 1:**
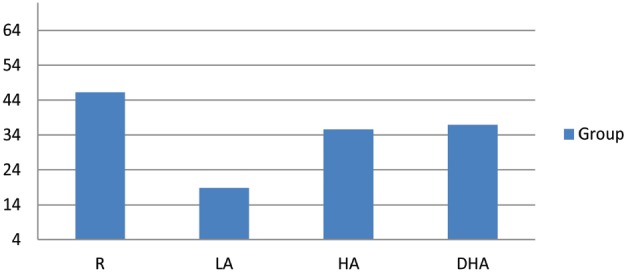
**Mean total OAS scores for R, LA, HA, and DHA**.

Table 1 shows means (and standard deviations) and effect sizes for OAS scores. As hypothesized, for overall OAS and all subscales R scored significantly higher than LA. R and the two high anxiety groups HA and DHA did not significantly differ on overall OAS and all subscales. Means of overall OAS are also shown in Figure 1. DHA scored significantly lower than HA on the distant subscale. Effect sizes were moderate.

**TABLE 1:** **Means (and standard deviations) for total Observer Alexithymia Scale (OAS) scores and subscales for repressor (R), low-anxious (LA), high-anxious (HA), and defensive high-anxious (DHA) groups. Effect sizes (r) are reported for predicted differences between R and LA**.

	**R**	**LA**	**HA**	**DHA**	**F (3,46)**	**Effect size (r)**
Distant	22.57_*a*_ (6.34)	10.73_*b*_ (7.72)	18.46_*a*_ (8.78)	15.70_*b*_ (8.83)	5.29***	0.72
Uninsightful	7.03_*a*_ (4.02)	2.88_*b*_ (3.20)	6.15_*a*_ (5.06)	6.85_*a*_ (6.32)	2.89*	0.55
Rigid	5.96_*a*_ (3.54)	1.53_*b*_ (1.91)	4.23_*a*_ (4.35)	4.8_*a*_ (4.20)	3.58*	0.71
Humorless	10.60_*a*_ (3.51)	3.73_*b*_ (4.10)	8.11_*a*_ (6.39)	8.25_*a*_ (4.99)	4.63**	0.67
Total OAS	46.28_*a*_ (13.19)	18.88_*b*_ (15.35)	35.6_*a*_ (21.34)	36.96_*a*_ (22.43)	5.28***	0.75

*p < 0.05, **p < 0.01, ***p < 0.001, Different subscripts indicate group differences (p < 0.05).

### TAS-20

**FIGURE 2:**
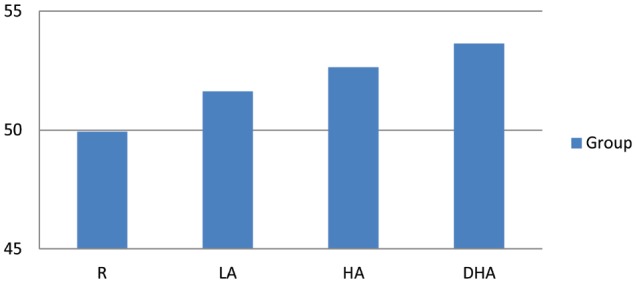
**Mean total TAS-20 scores for R, LA, HA, and DHA**.

As hypothesized, there was a significant main effect of Group, with R scoring significantly lower than all three control groups on the TAS-20 [R, mean = 40.93, SD = 5.84; LA, mean = 51.62, SD = 7.64; HA, mean = 52.64, SD = 4.73; DHA, mean = 53.46, SD = 10.54; *F*(3, 52) = 9.03, *p* < 0.001, *r* = 0.65]. Means of overall TAS are shown in Figure 2.

### Relationship between the TAS-20, OAS, Trait Anxiety (MAS), and Defensiveness (MC)

TAS-20 was not significantly correlated with total OAS or the subscales. However, for the individual groups, the TAS-20 and total OAS were significantly correlated for the HA group only (*r* = 0.41, *p* < 0.05). There was a significant correlation between TAS-20 and trait anxiety (*r* = 0.46, *p* < 0.0001) which was mainly driven by the two low anxiety groups for whom this relationship was significant (R, *r* = 0.55 *p* < 0.01, LA, *r* = 0.38, *p* < 0.05) and not the high anxiety groups (HA, *r* = 0.30, ns and DHA, *r* = 0.19, ns).

## Discussion

To the authors’ knowledge this is the first study to provide evidence that similar to anxious individuals, repressors score high on alexithymia on an independently-rated interview. This allows future studies to explore this link between the two constructs in more detail.

The current study demonstrated the predicted disassociation between an independently-rated interview measure and questionnaire measure of alexithymia, similar to previous findings on repressors’ childhood experiences (Myers and Brewin, 1994; Myers, 1999; Myers et al., 1999). These results also provide further evidence of repressors’ avoidance of negative information regarding the self: as consistent with previous studies a standard questionnaire measure indicated that repressors score low on alexithymia (Newton and Contrada, 1994; Myers, 1995). These results strengthen the conclusion that standard questionnaire measures are not suitable when investigating repressors (see Myers, 2000, 2010, for reviews). In future, questionnaires such as the TAS-20 should not be used in studies which investigate the repressive coping style and alexithymia.

However, previous reviews concluded that repressors’ avoidance of negative affect may be bypassed, as numerous studies indicate that repressors answer self-report measures in such a way to conclude that they avoid *negative* information regarding the self rather than being overly *positive* (see Myers, 2000, 2010). Firstly, evidence suggests that repressors tend to downplay the negative rather than overstating the positive (Myers and Brewin, 1996; Codd and Myers, 2009). Secondly, if repressors are allowed to express themselves positively on some items of a self-report measure they may rate themselves less positively on other items (Vetere and Myers, 2002). Thirdly, repressors may rate themselves negatively on some items but not on others (Myers, 1998). Fourthly, repressors may rate themselves differently on indirect measures compared with direct measures (Creswell and Myers, 2002). Therefore, a self-report measure could be developed specifically to assess alexithymia in future studies of alexithymia and repressive coping. This self-report measure should contain some items which allow repressors to express themselves positively so they may rate themselves less positively on other items and, where appropriate, could also require participants to give descriptions of specific events without allowing repressors to emotionally evaluate those events, which can be assessed by independent raters.

As discussed elsewhere (Myers, 2010), a substantial number of studies that identify the repressor group and control groups do not include a defensive high-anxious group, just the high-anxious, and low-anxious groups as controls (e.g., Dawkins and Furnham, 1989; Newton and Contrada, 1994; Frasure-Smith et al., 2002; Millar, 2006). The problem with this is that any group differences between repressors and controls could just be due to repressors high defensiveness scores alone and not due to their unique combination of low anxiety and high defensiveness. As the current study included the defensive high-anxious, it can be concluded that group differences on the TAS-20 were due to repressors unique combination of anxiety and defensiveness. There were no hypothesized differences between repressors and defensive high-anxious on the OAS, although it should be noted that the defensive high-anxious scored significantly lower than repressors on the distant subscale of the OAS.

In previous studies, the OAS has been rated by people who know the participants (e.g.,Berthoz et al., 2005, 2007; Lumley et al., 2005). In the current study, raters had not met the participants: they solely listened to an interview. This extends the use of the OAS and indicates that raters do not necessarily had to have met the participants.

There are some potential limitations to this study. The beth israel questionnaire (BIQ; Taylor et al., 2000) is a commonly used interview measure of alexithymia. However, the OAS was deemed more suitable than the BIQ for a number of reasons. Firstly, the BIQ is an interviewer-rated measure, not an instrument for independent rating, and this is reflected in the wording of some of the items, e.g., “has difficulty communicating his/her feelings *to me*.” Secondly, five items concern dreams, daydreams, fantasies and symptoms which were not part of the current study. The result was that out of 12 items only 6 could potentially be used because of the reasons outlined above.

In contrast, the OAS was designed “from the perspective of a collateral” (Lumley et al., 2005), i.e., it was not designed to be filled out by a clinician after a clinical interview. The current study involved listening to an interview which was not specifically designed to measure alexithymia which was comparable, as ratings would be collateral.

The OAS measures what Haviland et al. (2000) considered key elements of alexithymia: distant, uninsightful, somatizing, humorless, rigid. Thus it seemed more suitable to use this measure in our study as the majority of items could be scored by independent raters, e.g., “is a warm person.” Finally, we were able to rate the participants on 28 out of 33 items, only having to omit five items on the somatizing subscale which was not relevant to our main manipulations.

The interview did not have any information on physical health, so we could not rate the somatizing subscale. Also the sample was small, although the effect sizes were satisfactory and the numbers of participants are comparable with other repressive coping studies (Weinberger et al., 1979; Myers and Brewin, 1994; Derakshan and Eysenck, 1997; Lambie and Baker, 2003). These limitations should be addressed in future studies.

Future studies could use brain imaging to bypass repressors avoidance of negative affect, similar to Paul et al. (2012). In addition, studies have used brain imaging to investigate empathy and alexithymia (for a review see Bird and Viding, 2014). Similar brain imaging studies could be used to further investigate the relationship between alexithymia and repressive coping.

In summary, this study has identified a link between repressive coping and alexithymia. It has also highlighted the importance of using other measures rather than standard self-report measures to identify repressors, who make up to 50% of various populations.

## Author Contributions

LM conceived the study, designed the study, analyzed and interpreted the data, She drafted the paper and revised it critically for important intellectual content. She approved the final version and is accountable for all aspects of the work. ND helped to conceive the study, design the study and interpret the data. She made a substantial contribution in revising it critically for important intellectual content. She approved the final version and is accountable for all aspects of the work.

### Conflict of Interest Statement

The authors declare that the research was conducted in the absence of any commercial or financial relationships that could be construed as a potential conflict of interest.
